# Tailored cellulose nanocrystals as a functional ultraviolet absorbing nanofiller of epoxy polymers

**DOI:** 10.1039/c9na00265k

**Published:** 2019-05-20

**Authors:** Prachiben Panchal, Tizazu H. Mekonnen

**Affiliations:** Department of Chemical Engineering, University of Waterloo Waterloo ON N2L 3G1 Canada tmekonnen@uwaterloo.ca

## Abstract

Epoxy is an extensively used polymer in several applications such as coatings, adhesives, structural composites *etc.* However, it is a poor ultraviolet (UV) absorber and suffers from UV-degradation, which usually leads to discoloration and loss of structural integrity. In this study, cellulose nanocrystals (CNCs) conjugated with a UV absorbing molecule were investigated as a functional nanomaterial to enhance the UV absorption of epoxy polymers. The grafting of a UV absorbing molecule, *para*-aminobenzoic acid (PABA), on the surface of CNCs was confirmed using FTIR, proton NMR, and *via* elemental analysis. The modified CNCs were then incorporated into an epoxy polymer and their efficacy in mitigating the photo-degradation of epoxy was evaluated. For this, a neat epoxy control, native CNCs and modified CNC based nanocomposite specimens were subjected to controlled UV irradiation and the resulting structure–property changes were assessed. Results of UV absorption and discoloration showed that the neat epoxy was impacted the most as a result of the UV irradiation. While the incorporation of native CNCs displayed some UV absorption and reduction in the UV mediated discoloration of the epoxy polymer, the most pronounced effect was obtained in PABA decorated CNC based epoxy nanocomposites. The use of such tailored CNCs has great potential to mitigate UV induced degradation of a range of polymers that are used especially in outdoor applications where direct exposure to UV is significant.

## Abstract 2

Epoxy composites filled with UV filter decorated CNCs displayed remarkable structural stability and less discoloration under UV exposure.

## Graphical abstract

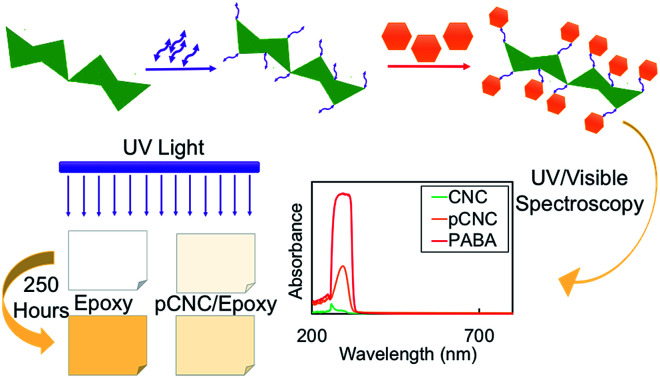

## 1. Introduction

Epoxy, a low molecular weight, thermosetting polymer resin, is extensively used in adhesives, paints and coatings, structural composites, electronics, construction and biomedical applications.^1^ The majority of these applications are found to be outdoors involving continuous exposure of the epoxy surface to sunlight. Along with a number of other radiations the sun emits ultraviolet (UV) rays, which are not only harmful to animals and plants but also damage materials such as plastics.^2,3^ While the UV rays with longer wavelengths such as UVA (315–400 nm) and UVB (280–325 nm) are absorbed by the ozone layer, some remaining UVB rays are transmitted to the earth with enough energy to break certain chemical bonds leading to accelerated chemical degradation of polymers.^2,4,5^ In the literature, the UV-degradation of polymers such as polyvinyl alcohol, polyurethane, and polyvinyl chloride has been reported.^2,6–8^ Despite its extensive use in outdoor applications, epoxy is among these polymers that undergo considerable photo-degradation under the exposure of ultraviolet (UV) light.^6,9,10^

Epoxies exposed to UV radiation undergo discoloration making them aesthetically unappealing.^6^ Longer exposure of epoxy to UV light also results in embrittlement of the polymer starting with the formation of micro-cracks on the surface.^6,10^ These are particularly unsuitable for epoxies used in coating or structural composite applications. Thus, epoxy applications in coatings are typically limited to underlayers that are not directly exposed to UV radiation. Furthermore, UV degradation deteriorates the mechanical properties of epoxy resins when used as a matrix in structural composites.^6^ Such a compromise in mechanical properties poses a disadvantage for epoxy *via* shortening its service life span in critical structural and functional applications such as in the automotive, construction, and aerospace industries. Hence, most epoxy composites used in structural material applications are coated with UV resistant polymers.

The incorporation of organic and inorganic UV absorbers and other stabilizers to polymeric material formulations has been researched and commercialized. The direct incorporation of inorganic UV stabilizers, such as ZnO and TiO_2_, and organic stabilizers, such as benzotriazoles, benzophenones, benzoic acid, and hindered amine light stabilizers (HALS) is extensively studied to reduce UV and other types of photodegradation.^5,9,11^ The use of *para*-aminobenzoic acid (PABA) as a UVB blocker in sunscreen lotions to prevent skin damage and in extreme cases skin cancer resulting from UV radiation is known to be due to its UV-blocking properties in the UVB range.^12^ While a few studies^2,13^ investigated the use of PABA as a potential UV absorber in polymers, its extensive use in the polymer industry is rather limited. For instance, Fernandes *et al.*^13^ grafted PABA to lignin and used the derivative as a co-blend with polyvinyl alcohol. Results of the studies showed that the lignin–PABA provided antioxidant/photo-protective properties to the PVA. However, it is worth mentioning here that lignin itself has UV absorption properties, and the observed photo-protection could have been from a combination of the lignin and the PABA.^14^

The direct incorporation of PABA as a UV absorber in polymers is also uncommon. This is attributed to its tendency of migration and decomposition that results in reduced efficiency over an intermediate to longer period of time.^11^ Moreover, such small organic molecules (MW 137.14 g mol^−1^) can easily leach out of plastics and may pose an environmental hazard.^2^ Particularly with regard to epoxy, PABA could not be directly incorporated in the baseline resin. This was because the highly nucleophilic primary amine group of PABA can open and react with the epoxide groups. This can severely limit the crosslinking of epoxy with the typical bifunctional amine crosslinking agent resulting in a less crosslinked network with poor properties. In this research, the grafting of PABA on the surface of cellulose nanocrystals (CNCs) and its UV absorption efficiency in epoxy nanocomposites were investigated. The covalent bonding of PABA on the surface of CNCs can circumvent the problem of leaching from polymer matrices over the lifetime of the polymer applications and potentially provide a reinforcing effect. Also, CNCs have already attracted substantial interest as a reinforcing filler of polymers, and their use as a multifunctional filler could be of great scientific and industrial interest.

Cellulose nanocrystals (CNCs) were selected as model nanoparticles due to their substantial importance in the polymer industry associated with their renewability, biodegradability, high strength, and low density. Moreover, CNCs are rich in hydroxyl groups associated with their anhydrous glucose monomer units. These hydroxyl moieties, typically one primary and two secondary, are amenable to several chemical modifications.^15,16^ A significant body of research has already been conducted on the use of CNCs as a reinforcing filler of epoxy polymers.^17,18^ Investigation of the utilization of nanoparticles and other organic molecules to mitigate UV degradation of polymers is not entirely new as well. For instance, Woo *et al.*^6,10^ have studied the photo-degradation and resulting performance of epoxy resins with organoclay as a filler. Nikafshar *et al.*^19^ used an organic filler (Tinuvin 1130) to enhance the UV resistance of epoxy. Despite the diversity of modification chemistries and the promising properties already offered by functional agents grafted onto CNCs, the full potential of CNCs has not been utilized, and more applied approaches are in great demand. Furthermore, the use of coupling pathways to attach functional molecules onto CNCs is still relatively rare, most likely due to the lack of facile conjugation agents and methods. CNCs modified with PABA can be used as a filler for polymeric matrices to reduce degradation caused by extensive exposure to UV-light. Thus, the goal of this study was to investigate the conjugation of PABA onto CNCs using a coupling agent that has a differentiated reactivity and evaluate their efficiency as a functional UV protective nanomaterial in epoxy matrices.

## 2. Experimental

### 2.1. Materials

Spray dried cellulose nanocrystals (CNCs) produced *via* sulfuric acid hydrolysis of bleached kraft pulp with a crystallinity of 88% were obtained from CelluForce Inc. (Montreal, Canada). Isophorone diisocyanate (IPDI), dimethyl sulfoxide (DMSO), dibutylin dilaurate (DBTDL), bisphenol A diglycidyl ether epoxy resin, poly(propylene glycol) bis(2-aminopropyl ether) curing agent, toluene, and acetone were purchased from Sigma Aldrich. 4-Aminobenzoic acid (PABA) was obtained from Fisher Scientific. All the chemicals used in this research were of analytical grade and used as received unless otherwise mentioned.

### 2.2. Preparation of modified CNCs

The grafting of PABA to the CNCs was conducted in a two-step heterogeneous reaction process. In the first step, 2% (w/v) of dried CNCs was dispersed in DMSO *via* magnetic stirring followed by homogenization (Homogenizer, PowerGen 700) for a total of 15 min and heated to 60 °C. Concurrently, the IPDI coupling agent was heated to 60 °C and 0.8 mol% DBTDL catalyst was added and stirred for 5 min in a nitrogen atmosphere. The pre-heated IPDI/DBTDL and CNC dispersions were then mixed at a weight ratio of 1 : 30 (CNC : IPDI/DBTDL) in a glass reactor equipped with a stirrer and thermostat. The reaction was then carried out at 60 °C under a nitrogen atmosphere for 4 hours. The CNC–IPDI intermediate product was then washed with toluene, which involved dispersion in toluene and centrifugation (4000 rpm for 3 min), which was repeated five times to remove unreacted IPDI. The recovered CNC–IPDI intermediate is referred to as iCNC throughout the manuscript. One portion of iCNC was oven dried at 80 °C overnight for further characterization.

The remaining iCNC was re-dispersed in DMSO and mixed with the UV absorbing molecule, PABA, which was solubilized in DMSO and pre-heated to 60 °C to produce the final tailored CNC nanoparticles. The weight ratio of CNCs to PABA in this reaction was set to 1 : 3 based on preliminary experimental observation, and the reaction was conducted at 60 °C overnight. The product was recovered *via* successive toluene and acetone wash. This involved dispersing the product in excess toluene followed by centrifugation (4000 rpm for 3 minutes) three times followed by acetone washing (three times). The washed final product (CNC–IPDI–PABA) referred to as pCNC throughout this manuscript was dried overnight at 80 °C and used.

### 2.3. Characterization of the modified CNCs

#### 2.3.1. Fourier Transform Infrared Spectroscopy (FTIR)

In order to investigate the modification of CNCs, samples were characterized by Fourier Transform Infrared Spectroscopy (FTIR) using a Nicolet 6700 model, Thermo Scientific unit. Sample pellets were prepared by mixing dried sample powders of native CNCs, iCNC, pCNC, PABA, and nanocomposites with dried potassium bromide (KBr) salt powder and pressing them into pellets using a Carver press. FTIR scans, in transmittance mode, were then collected in the frequency range of 500 and 4000 cm^−1^.

#### 2.3.2. Elemental analysis

Elemental analysis was carried out to confirm the coupling of IPDI to cellulose nanocrystals to produce the IPDI–CNC (iCNC) intermediate product, followed by attachment of PABA to the iCNC to produce PABA–IPDI–CNC (pCNC). CNC, iCNC and pCNC samples were dried overnight at 70 °C prior to analysis. The elements C, H, and N were quantified using a 4010 elemental analyzer (Costech instruments, Italy) equipped with a Delta Plus XL continuous flow isotope ratio mass spectrometer (Thermo-Finnigan, Germany).

The coupling efficiency of PABA onto CNCs was calculated in accordance with a method reported by Guan *et al.*^20^ based on elemental analysis results. The coupling efficiency was calculated as:1
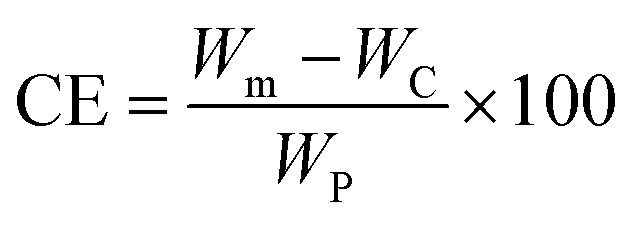
*W*_m_, *W*_C_, and *W*_P_ represent the weight of modified CNCs (pCNC), native CNCs and PABA, respectively. *W*_C_ and *W*_P_ were the weights used to conduct the reaction, and *W*_m_ was a calculated value as shown below.2
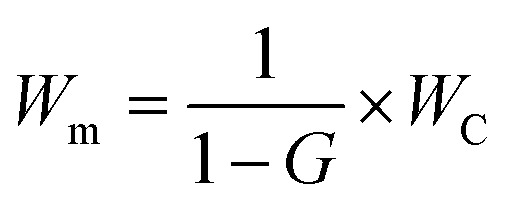
3
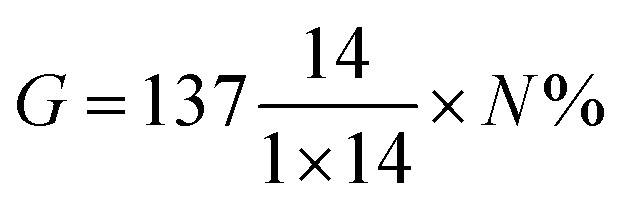
*G* is the weight of PABA in the modified CNCs, which was calculated based on the percent nitrogen (*N*%) obtained from elemental analysis. 137.14, 1 and 14 represent the molar mass of PABA, number of nitrogen atoms present in PABA, and molar mass of nitrogen, respectively. The calculated weight of PABA in pCNC (*G*) was then used to calculate *W*_m_.

#### 2.3.3. Proton nuclear magnetic resonance (^1^H NMR)

The proton NMR spectra of CNCs, PABA, iCNC and pCNC were recorded using a Bruker 500 MHz high-resolution NMR spectrophotometer (Bruker-electrospin 500 MHz Ultrashield, Bruker Corporation, MA). Samples were prepared by dispersing 1 mg in deuterated DMSO and the spectra were collected at 500 MHz at room temperature.

#### 2.3.4. Thermogravimetric analysis (TGA)

The thermal stability of CNCs, modified CNCs, and their nanocomposites was evaluated using a TA instrument (TGA Q500) to study the impact of modification on the thermal behavior of CNCs. The thermal degradation behaviour of the samples was recorded from 25 °C to 550 °C with a ramp rate of 5 °C min under a nitrogen environment. The weight loss was used as an indicator to analyze the thermal stability/degradation behaviour of the samples.

#### 2.3.5. Contact angle measurement

The impact of attaching PABA on the hydrophilicity of CNCs was studied by employing water contact angle (WCA) measurement. In order to test the WCA, 200 mg of CNCs and pCNC were pressed into two pellets using a Carver press. The pellets were then dried at 60 °C for an hour before conducting the contact angle measurement. Using a sessile drop device, a water droplet was dropped onto the pellets, and the instantaneous water contact angle was recorded.

#### 2.3.6. UV-visible (UV-vis) spectroscopy

The UV absorbance of native CNCs, modified CNCs, PABA, and nanocomposite samples was analyzed using a Cary 100 Bio UV-visible (UV-vis) spectrophotometer. For this, 1 g of CNCs and pCNC were dispersed in 10 mL of DMSO, and the UV absorbance was collected from 200 to 800 nm wavelength with DMSO as the baseline solvent. Similarly, the effect of the UV absorber (grafted onto the CNCs) on the epoxy–CNC nanocomposites was analyzed using a UV-vis spectrophotometer. A neat epoxy polymer that was not exposed to UV irradiation (Neat epoxy_0) was used as the baseline material and other samples with varying CNC and pCNC compositions and UV irradiation treatments were analyzed from 200 nm to 800 nm.

### 2.4. Epoxy–CNC nanocomposites

#### 2.4.1. Preparation

Epoxy matrices for the nanocomposite sheets were prepared by combining 70% (w/w) epoxy resin and 30% (w/w) curing agent. Nanocomposites were prepared by incorporating 5 wt% and 10 wt% loading content of native and modified CNCs (pCNC). For this, calculated quantities of CNCs and pCNC were first dispersed well in the amine based curing agent using a homogenizer (PowerGen 700) and then mixed with the epoxy resin. The mixture was then further sonicated using an ultrasonic processor (FB120, Fisher Scientific) to obtain an enhanced dispersion of the native and tailored CNCs, and degassed to remove air bubbles. Resin mixtures prepared as such were then poured into a silicon mold and cured at 60 °C for four hours followed by room temperature curing overnight to produce the nanocomposite test specimens.

#### 2.4.2. UV-irradiation

Epoxy nanocomposites, which include neat epoxy baseline, epoxy–CNCs and epoxy–pCNC, were subjected to a controlled UV irradiation exposure. For this, a UV lamp obtained from LSE Lighting (120 V and 50 W) was placed 25 cm above the samples and allowed to irradiate the samples for 0 (control), 72 and 144 hours, and in the case of selected samples for 250 and 500 hours. For labelling purposes, a three part system was used, *xy*_*z*. The prefix *x* represents the weight percent of the filler added to epoxy composites. Epoxy composites were prepared with different filler loadings indicated by *x* (5 and 10 wt%), and *y* is the filler type used (CNCs, pCNC and PABA). Finally, *z* is the number of hours the sample was exposed to UV irradiation (0, 72, 144, 250 or 500 hours). The epoxy without any filler added was denoted as neat epoxy with appropriate irradiation time.

#### 2.4.3. Color analysis

Discoloration is one of the indications of photo-degradation. In order to quantify the change in color for UV irradiated epoxy nanocomposites, samples were tested using a MiniScan EZ Diffuse SAV color measurement spectrophotometer (HunterLab, VA, USA). Samples were tested at least in triplicate, and the average was used for further calculations. L*a*b* values were measured, which represent the lightening–fading, red–green and yellow–blue coordinates, respectively.^21^ A positive ΔL* value represents lightening of the samples and a negative value represents fading. Similarly, a positive Δa* corresponds to a color shift towards red, and negative, towards green. In this way, a positive Δb* corresponds to a color shift towards yellow, and a color shift towards blue is represented by a negative Δb*. The total change in color was then computed using the following expression:4
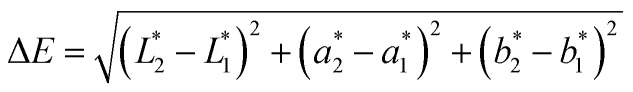


The subscripts 1 and 2 correspond to the values for the reference sample and values for samples after exposure, respectively. For each of the epoxy nanocomposites, samples before exposure, 0 hours of exposure, were taken as reference samples to study the discoloration effect due to UV exposure.

#### 2.4.4. Water absorption

The moisture absorption of the baseline epoxy, native CNCs, and modified CNC based nanocomposites was studied in accordance with ASTM D570-98. Sample specimens were cut into 20 × 20 × 2 mm pieces and dried in a vacuum oven at 60 °C for two hours. The weight of each dried sample was recorded as *W*_1_ and then they were submerged in deionized water at room temperature. After 24 hours the samples were removed from water, their surface dried with a piece of blotting paper, and weighed, which was recorded as *W*_2_. The samples were then re-immersed in water and the process was repeated for 7 days with measurement every 24 hours. The water absorption, which was measured in terms of weight increase, was calculated using the following relation:5
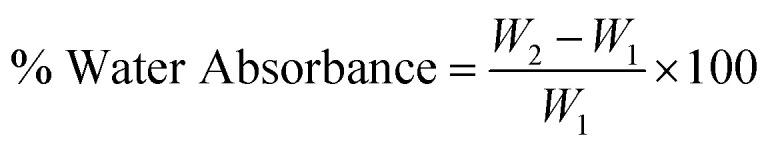


### 2.5. Statistical analysis

Experimental replicate results were expressed as the mean value ± standard deviation. The statistical analyses of the data were conducted using the statistical software package Minitab (Version 18). Single factor analysis of variance (ANOVA) was employed to identify significant differences among mean values with a 95% confidence level (*P* < 0.05) of LSD criteria.

## 3. Results and discussion

### 3.1. Modification of CNCs

#### 3.1.1. Surface grafting of PABA on CNCs

**Scheme 1:**
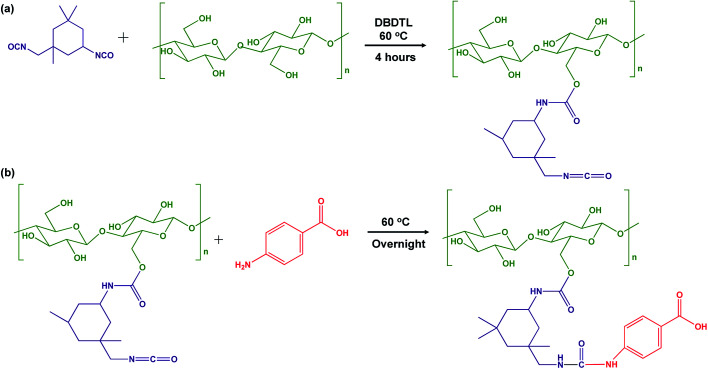
Reaction scheme of (a) modification of CNCs surface using IPDI to produce CNC–IPDI intermediate product (iCNC); (b) attachment of PABA to modified CNCs to produce CNC–IPDI–PABA (pCNC).

The surface of cellulose nanocrystals was decorated with PABA, a UV absorbing molecule, using IPDI as the coupling agent. The grafting of PABA onto CNCs was conducted in a two-step process as shown in Scheme 1. IPDI, containing dual isocyanate functional groups with differentiated selectivity, was used as a coupling agent between PABA and the CNC nanoparticles. It has been reported that in the presence of DBTDL as the reaction catalyst, the secondary isocyanate functional group of IPDI has higher reactivity than the primary group.^22,23^ In Scheme 1(a) the first step of the reaction is presented, where the secondary isocyanate group was activated in the presence of DBTDL at a moderate temperature (60 °C). The isocyanate group (–NCO) was then covalently bonded with hydroxyls (–OH) on the surface of CNCs as reported by Girouard *et al.* producing the intermediate product CNC–IPDI (iCNC).^23^ The primary amine group of PABA was then allowed to react with the unreacted primary–NCO group of the iCNC intermediate product as shown in Scheme 1(b) to produce CNC–IPDI–PABA (pCNC). To validate these modifications of CNCs, FTIR, elemental analysis, and ^1^H NMR analysis were employed and discussed in the next sections.

**Fig. 1:**
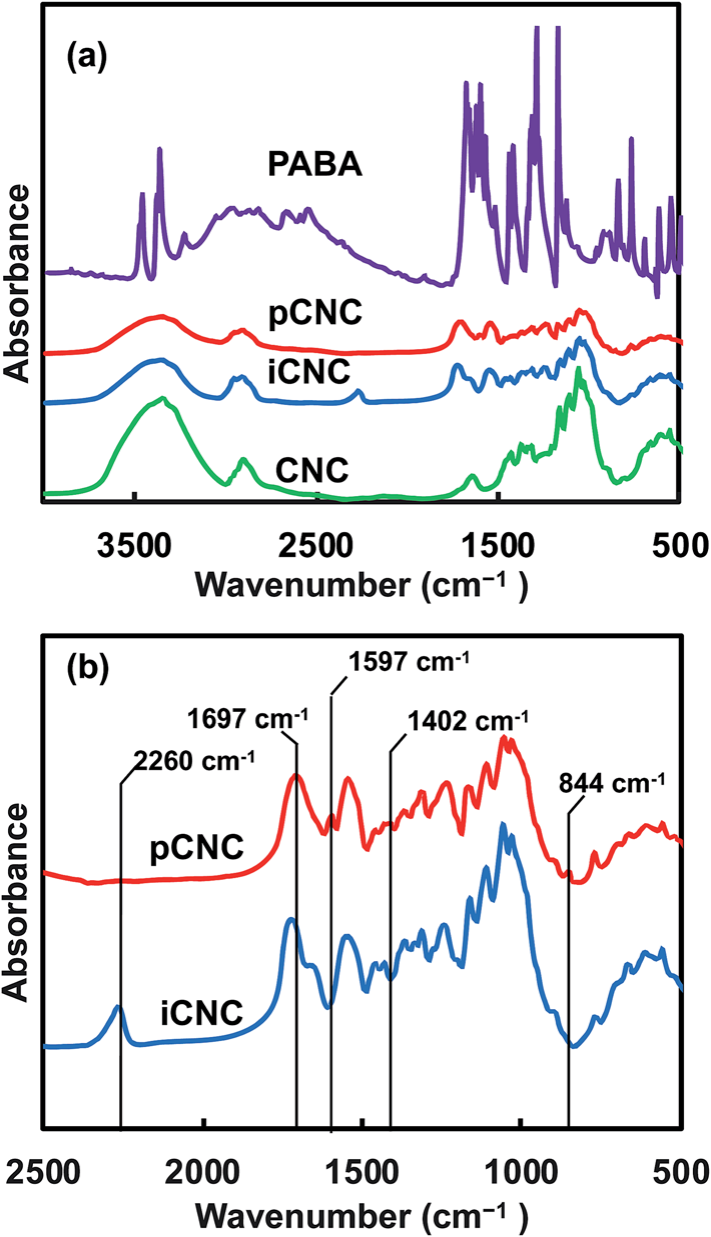
(a) IR spectra of CNCs, iCNC, pCNC, and PABA. (b) Detailed spectra of the intermediate (iCNC) and final reaction product (pCNC).

Native and modified CNCs, iCNC, pCNC and PABA were analyzed using IR to confirm the proposed grafting between PABA and the CNCs. Fig. 1(a) shows the comparison of the IR spectra for CNCs, iCNC and pCNC along with those of PABA. The broad peak at 3000–3550 cm^−1^of the CNCs was attributed to –OH functional groups. The reduction in the –OH peak (3000–3550 cm^−1^) and appearance of the –NCO peak at 2240 cm^−1^ for iCNC as shown in Fig. 1(a) were indicative of the modification of surface hydroxyls on CNCs as illustrated in Scheme 1(a) to form iCNC.^23–25^ Furthermore, the emergence of a carbonyl band at 1700 cm^−1^ in iCNC provided further evidence of urethane linkage formed as –NCO reacted with the –OH moieties of CNCs.^26,27^

The reaction between the amine groups of PABA and the unreacted –NCO of the intermediate product as shown in Scheme 1(b) was observed from the complete consumption of the –NCO in pCNC compared to that in iCNC (Fig. 1(a)). Close analysis of pCNC (Fig. 1(b)) showed the presence of an absorbance band at 1597 and 1402 cm^−1^, which were attributed to the C=C bond from the aromatic ring of PABA.^28^ The –C–H bond of the benzene ring, observed at 844 cm^−1^, was additional evidence of a conjugated PABA onto the CNC surface. Moreover, the broadening of the carbonyl peak at 1697 cm^−1^ in pCNC compared to that in iCNC was indicative of further modifications. The carboxyl group from PABA and conjugation of the primary and secondary isocyanate group leading to urea and urethane linkages, respectively, were detected with the carbonyl peak observed at 1775–1625 cm^−1^ with the maxima at 1697 cm^−1^.^28,29^

**Fig. 2:**
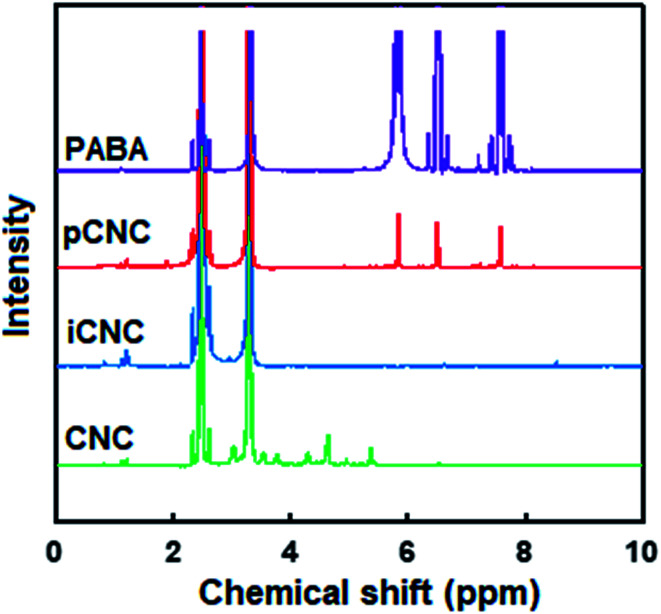
Proton NMR spectra analysis of PABA grafting onto CNCs.

The tailoring of CNCs was further analyzed by ^1^H NMR. Fig. 2 displays the proton signal spectrum of the different samples. The main peaks at *δ* 2.51 and 3.33 ppm in all the samples correspond to DMSO, the solvent in which the samples were dispersed to obtain the ^1^H NMR spectra. Characteristic signals of CNCs chain backbone was observed at *δ* 3.6, 3.9, 4.7 and 5.48 ppm.^2^ The ^1^H NMR spectrum for PABA showed the aryl protons at *δ* 5.94, 6.57 and 7.53 ppm. The protons adjacent to amine (–NH_2_) on PABA cause the signal at 6.57 ppm to split into a triplet. As the amine group reacts with the isocyanate group in pCNC, a doublet was observed instead of a triplet at 6.57 ppm as one of the protons gets consumed in coupling. Substituents of the aromatic ring from the PABA in pCNC at 5.94, 6.42 and 7.61 ppm provided further confirmation of the covalent grafting of PABA onto CNCs.

In addition to FTIR and ^1^H NMR studies, the changes in elemental carbon and nitrogen composition between the native (CNCs), the reaction intermediate (iCNC) and final product (pCNC) samples were analyzed using elemental analysis. CNCs, a polymer of anhydrous glucose units, do not contain any nitrogen atoms. The increase in the nitrogen content for iCNC shown in Table 1 was attributed to the covalent attachment of isocyanate from IPDI. A further increase in nitrogen weight percent (6.4%) in pCNC was associated with the attachment of PABA to iCNC, and this is statistically significant (*p* < 0.05). Furthermore, the nitrogen weight percent from elemental analysis was used to estimate the coupling efficiency for modified CNCs according to Guan *et al.*^20^ Calculations based on eqn (1) provided a coupling efficiency of 8%. This demonstrated that the use of IPDI as a coupling agent allowed to graft sufficient amount of PABA onto the CNCs.

**Table 1:** Elemental analysis for the modification of CNCs^a^

Sample	%C	%N
CNCs	42.47 ± 1.08^a^	—
iCNC	48.93 ± 0.92^b^	5.47 ± 1.23^a^
pCNC	49.46 ± 2.75^b^	5.82 ± 1.03^a^

a Value – mean ± standard deviation (*n* = 3), means with the same superscript letters within a column are not significantly different at the *P* < 0.05 level.

#### 3.1.2. Characterization of modified CNCs

**Fig. 3:**
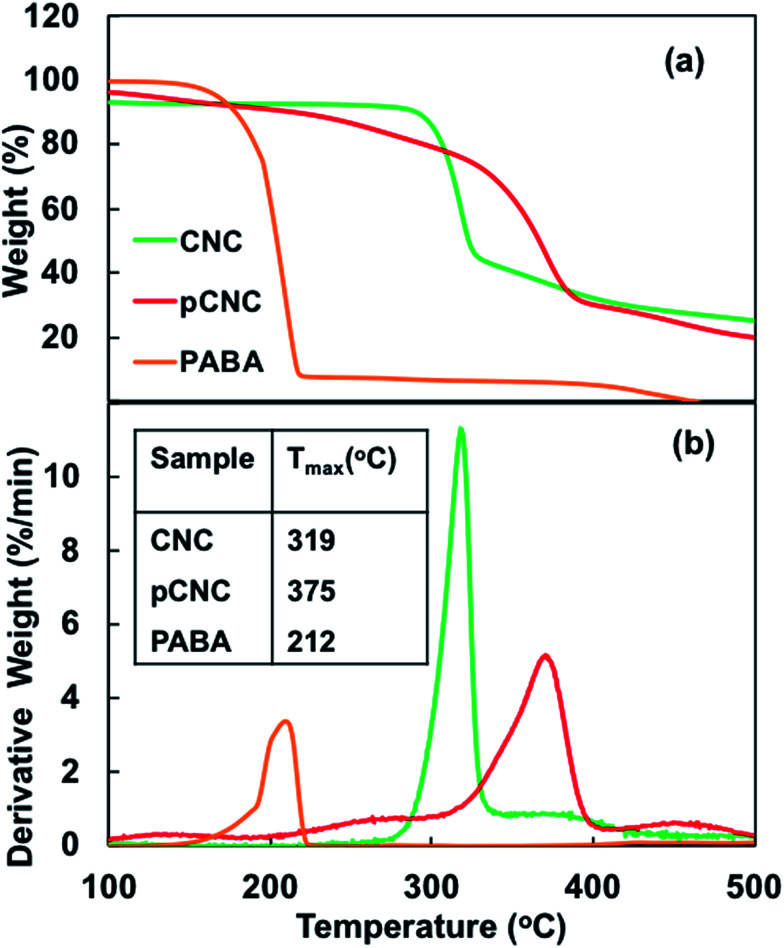
TGA analysis of CNCs, PABA and pCNC: (a) weight loss curve and (b) weight loss derivative curve.

The thermal stability of additives and fillers used in polymeric materials is important, because most polymer processing operations such as melt processing, thermal curing, and injection or compression molding typically involve high temperature. To evaluate the impact of grafting PABA onto CNCs on the thermal stability, native and modified CNCs (pCNC), and PABA were analysed using TGA, and their thermograms are shown in Fig. 3. The native CNCs and pCNC showed a slight weight loss near 100 °C which was attributed to the evaporation of bound water. A major weight loss of CNCs was observed between 295 and 325 °C with a degradation peak at 319 °C. The degradation of CNCs could be attributed to concurrent dehydration, depolymerisation of the cellulose chain, and decomposition of glycosyl units.^30^ As displayed in Fig. 3(b), the peak degradation temperature of PABA was observed at 211 °C, which was much lower than that of the native CNCs. In comparison to CNCs, the degradation of pCNC (PABA–CNC) began at a much lower temperature, with a 15% weight loss observed at 265 °C. This was likely associated with the degradation of the less thermally stable PABA that was attached to the surface of CNCs. Despite the lower thermal degradation initiation of the pCNC, the peak degradation was remarkably higher than that of CNCs (18% increase). The increase in the peak degradation temperature of pCNC beyond that of CNCs could be pertaining to the reduction in the number of surface hydroxyls groups, responsible for the thermal dehydration as a result of the grafting of PABA. This clearly indicated that the incorporation of PABA onto CNCs improved the thermal stability of native CNCs. Such an improvement in the thermal stability of CNCs is of great importance for their use as a functional filler in polymer nanocomposites as this limits the risk of degradation, which could occur during melt processing (in thermoplastics) or curing in thermoset polymers.

CNCs display great potential as a sustainable nanomaterial for a range of material applications. Studies have indicated that they show inherently high hydrophilicity owing to the tendency of their –OH moieties to associate with water *via* hydrogen bonding with slight variation depending on the source and extraction process of CNCs.^17^ The recorded water contact angle (WCA) of native CNCs ranges from 9–30°.^17,31–33^ This can be an advantage in some cases, for instance, to conduct water based chemistries and in certain applications that enjoy wettability and water dispersability.^34^ However, such high hydrophilicity is detrimental in many polymer nanocomposite systems, including epoxy mainly because of their lack of dispersibility in non-polar polymer systems. Some outstanding properties of the epoxy polymer that make it the polymer of choice in several applications can negatively be impacted *via* water absorption.^35–38^ For example, moisture uptake can cause a marked reduction in the modulus, toughness, crack resistance, and adhesion strength of epoxy materials.^38–40^ Combining the hydrophilic CNCs with epoxy is anticipated to aggravate the overall moisture absorption of the nanocomposites.

**Fig. 4:**
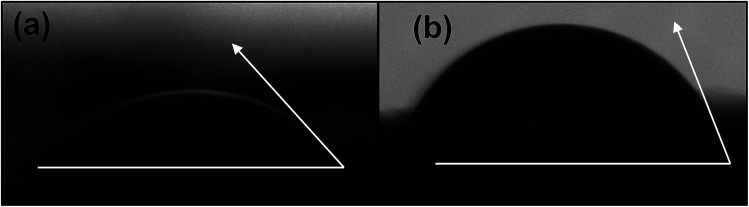
Water contact angles for (a) native CNCs and (b) pCNC (CNC–IPDI–PABA).

Fig. 4(a) and (b) present the WCA for native and modified CNCs. The WCA observed for native CNCs was 32.7°. CNCs modified with PABA showed a higher WCA (64.9°), which was a 98% increase from that of the native CNCs and a marked reduction in hydrophilicity. This was because the grafting of PABA onto CNCs consumed the hydroxyl groups responsible for the hydrophilicity of CNCs as shown in Scheme 1 and Fig. 1. Thus, the grafting of PABA onto CNCs significantly improved the hydrophobicity of CNCs, which is a desired attribute for enhancing their dispersibility in mostly non-polar polymers.

### 3.2. UV absorption

**Fig. 5:**
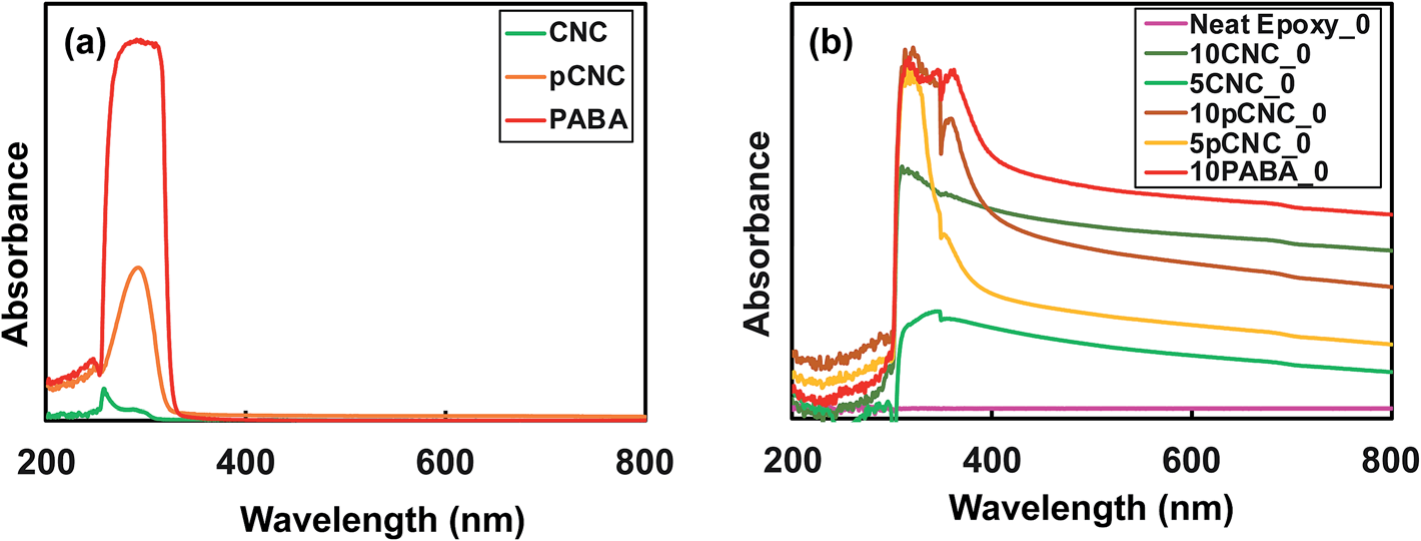
Comparative UV absorbance of (a) CNCs, PABA and pCNC; (b) epoxy nanocomposites prior to UV irradiation.

Epoxy resins are among the most common thermosetting polymers with a range of applications due to their marked physical properties, chemical resistance, and processability. However, epoxy based materials often degrade under direct exposure to UV radiation, which curtails their use in many applications. For instance, discoloration, chalking, loss of adhesion, cracking *etc.* as a result of direct UV exposure limit their use as an external layer coating in the construction and automotive industries.^41^ This is because epoxy polymers have an aromatic backbone chain that can absorb UV and undergoes detrimental chemical and physical changes, resulting in poor material performance properties.^42^ The incorporation of UV absorbing molecule decorated CNCs into epoxy and the UV absorption properties are investigated using a UV vis spectrophotometer and the results are presented in Fig. 5.

It was noted that native CNCs absorbed very minimal UV radiation. PABA on the other hand displayed excellent UV absorption within the UV light range, 250–340 nm. The CNCs with grafted PABA (pCNC) exhibited UV absorbance within a similar UV range (250–330 nm) to that of PABA. Compared to PABA lower absorbance was observed for pCNC, and this was because only 8% of PABA was attached to the CNCs. Despite the pCNC carrying a lower amount of PABA, it displayed magnificent UV absorbance properties, which can bring about the desired UV absorption when incorporated in polymer matrices.

As presented in Fig. 5(b), the spectra for neat epoxy showed no absorbance in the ultraviolet or visible region. This is in agreement with the study reported by Yuan-Qing *et al.*^43^. Epoxy–CNC nanocomposites showed slight UV absorbance, and it was evident that an increase in the CNC loading increased the UV absorbance when comparing the UV absorbance of 5CNC_0 with 10CNC_0 (Fig. 5(b)). However, the UV absorbance was enhanced significantly with the use of pCNC. For comparison reasons, 10 wt% PABA was directly incorporated in epoxy to produce 10PABA_0 epoxy nanocomposites that displayed the greatest UV absorbance. 10PABA_0 also showed an additional peak at 360 nm (in the visible range). A similar peak was observed for 10 wt% pCNC in epoxy. Comparing the direct addition of PABA in epoxy with pCNC, it was observed that the use of pCNC at both 5 and 10% loading displayed excellent UV absorption similar to that of the direct addition of PABA confirming that the anchored PABA onto CNCs maintained its UV absorbance efficiency. As expected, 10pCNC provided enhanced UV absorbance compared to 5pCNC. While the native CNCs exhibited some UV absorbance, the pCNC displayed remarkably higher UV absorbance owing to the aromatic benzene with carboxyl and amine functionalities of PABA that are known to provide high UV radiation absorption (290–320 nm in the UVB region).^44^

**Fig. 6:**
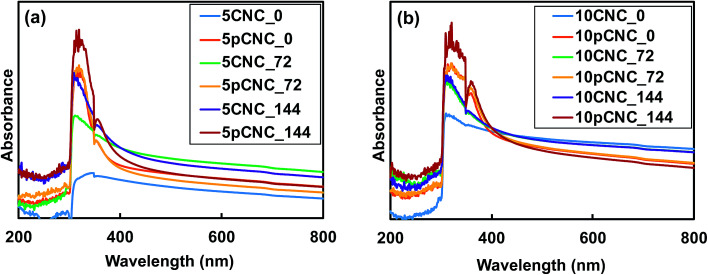
Effect of UV irradiation on the UV absorption of (a) epoxy nanocomposites with 5 wt% CNCs and pCNC after UV irradiation for 0, 72, and 144 hours; (b) epoxy nanocomposites with 10 wt% CNCs and pCNC after UV irradiation for 0, 72, and 144 hours.

Baseline epoxy, native CNCs and pCNC based epoxy composites were subjected to controlled UV irradiation for 72 and 144 hours, and the impact of the irradiation on the UV absorption properties and color changes was evaluated. Fig. 6(a) and (b) display the UV absorption of nanocomposite samples of CNCs and pCNC before and after UV-irradiation with 5 wt% and 10 wt% loading, respectively. It was observed that the nanocomposite samples with pCNC had greater absorbance than those that contain CNCs even after the UV irradiation. Moreover, as the samples were irradiated for a longer time (144 hours), the absorbance displayed a higher intensity, indicating that a continued UV exposure may not change the UV absorbance properties. Nikafshar *et al.*^5^ also reported a similar trend in which the UV absorbance of epoxy increased with an increase in the UV irradiation when another UV absorber (Tinuvin 1130) was used. A possible explanation for this observation could be that under UV-exposure molecular arrangements of nanocomposites occurred that led to the formation of chromophores which could absorb specific wavelengths resulting in higher absorbance.

PABA as a direct UV absorbing molecule in epoxy could not be used. This was because it has a primary amine group with high nucleophilicity that opens the epoxide ring and reacts with the epoxy resin. Such reactions reduce the reaction between the epoxy resin and the polyetheramine crosslinking agent resulting in a poorly crosslinked epoxy thermoset. Such reduced crosslinking density typically causes undesirable property deteriorations in the epoxy thermosets.^45,46^

### 3.3. Effect of UV irradiation on the chemical structure of epoxy nanocomposites

**Fig. 7:**
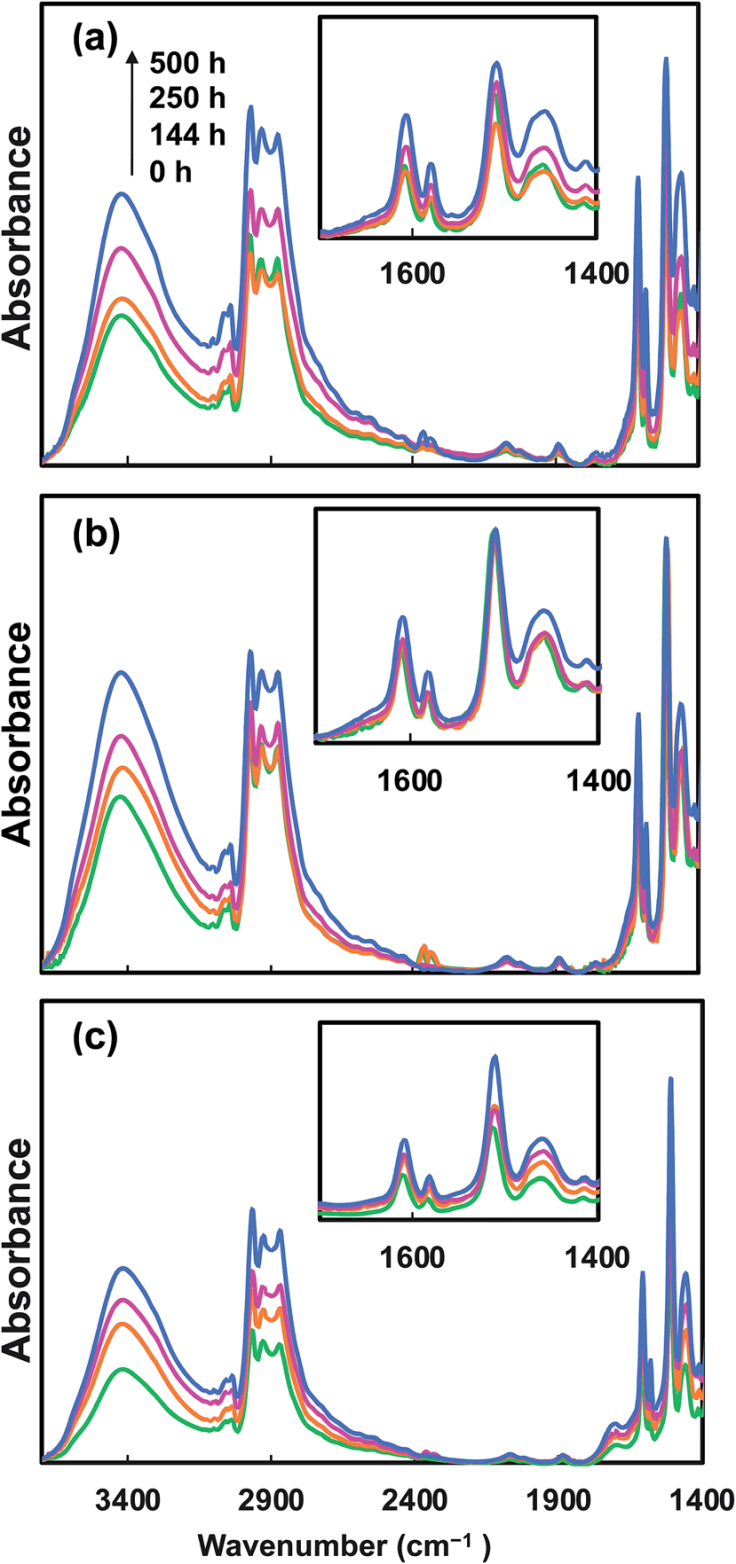
FTIR spectra of (a) neat epoxy, (b) 10CNC and (c) 10pCNC upon UV degradation.

The changes to the chemical structure of epoxy caused by UV irradiation were analyzed by FTIR, as epoxy goes through photo-degradation mainly through hydroxyl and carbonyl group formation. Such photo-degradation mechanisms of epoxy have been reported extensively in the literature.^9,19,47,48^ It has been reported that under extensive UV light exposure, oxidation of the phenoxy part of epoxy leads to the formation of hydroxyl and carbonyl groups.^9,19^ Furthermore, photo-reaction of hydroxy-alkyl-ether groups of epoxy forms methyl-end groups.^19,49^ In order to study the structural change of the nanocomposites, FTIR analysis of samples irradiated for different times was conducted. Fig. 7 presents the effects of such UV irradiation on the chemical structure of the epoxy nanocomposites. With an increase in the UV exposure time, all nanocomposites (neat epoxy baseline, 10CNC and 10pCNC) exhibited common changes as noted from the IR spectra. There was an increase in the hydroxyl groups between 3000 and 3650 cm^−1^ of the nanocomposites indicating oxidation of the phenoxy part. A remarkable increase in the peak between 2798–2985 and 1445 cm^−1^, which represents stretching of the C–H bond and the presence of methyl groups was noted as well. These can be attributed to the increase in the methyl groups formed as a result of reaction of hydroxy-ether groups. Moreover, an increase in the carbonyl peak at 1610 cm^−1^ was observed for all the nanocomposites. For the neat epoxy baseline another peak was observed at 1505 cm^−1^, which was intensified with an increase in the UV irradiation time. This can be attributed to the formation of chromophores, such as C=C. The formation of C=O and C=C was a result of chain scission or chain crosslinking.^10,48^ These were chromophoric chemical species, in which electrons were excited from the ground state upon absorbance of the light when exposed to UV light, leading to discoloration of epoxy materials.^10,48^

### 3.4. Discoloration of epoxy nanocomposites

**Fig. 8:**
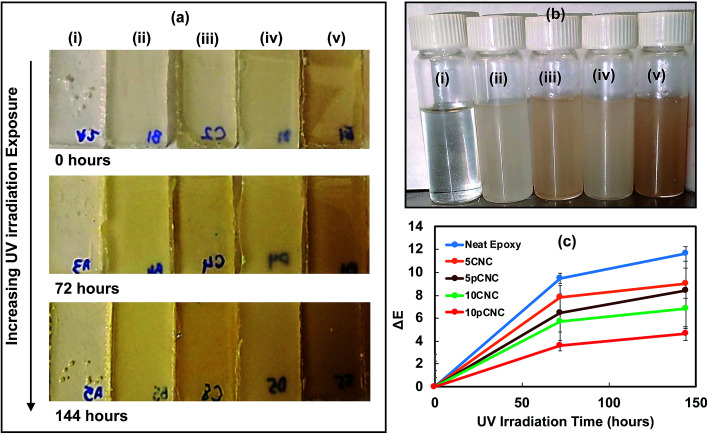
(a) Epoxy nanocomposites cured and UV irradiated for 0, 72 and 144 hours; (b) epoxy nanocomposites before curing: (i) neat epoxy, (ii) 5CNC, (iii) 5pCNC, (iv)10CNC and (v) 10pCNC, respectively; (c) color change related to samples.

One of the vital indicators of epoxy degradation is its discoloration. As epoxy is exposed to UV-light from the sun it displays yellowing over a period of time, making it aesthetically unappealing and leading to weakening of certain performance properties. Fig. 8(a) shows the appearance of the cured epoxy nanocomposite samples upon UV exposure. The top row shows the cured samples prior to UV-exposure (*t* = 0). The second and third row display the changes in the color of the nanocomposites as a result of UV exposure for 72 and 144 hours, respectively. The incorporation of both native CNCs and pCNC provided various degrees of off-colors to the transparent baseline epoxy as noticed from Fig. 8(a) (first row) and 8(b). The off-colors produced by pCNC were more pronounced. This was associated with the inherent grey color of the PABA molecule. As observed from Fig. 8(b), these off-colors were produced prior to curing.

The UV irradiation for 72 and 144 hours caused substantial color changes compared to the samples that were not exposed to UV irradiation. The color changes as a result of the UV irradiation were calculated based on colorimetric measurements and the results are shown in Fig. 8(c).

For the calculation, respective samples before UV irradiation were used as reference samples, thus, for each sample at 0 hours the calculated change was taken as the baseline. It can be noticed that as the time of irradiation increased more changes in color were observed in all nanocomposite samples. Neat epoxy was the most impacted as a result of the UV irradiation that led to extensive discoloration of the samples. As observed from Fig. 7, UV irradiation leads to the formation of chromophores, which may absorb visible light leading to discoloration. While the incorporation of native CNCs reduced the discoloration at both 5 wt% and 10 wt% loading concentrations, 10 wt% provided a more distinct reduction in discoloration compared to the non-filled epoxy baseline (Fig. 8(c)). However, the best results were achieved with the use of 10pCNC.

**Fig. 9:**
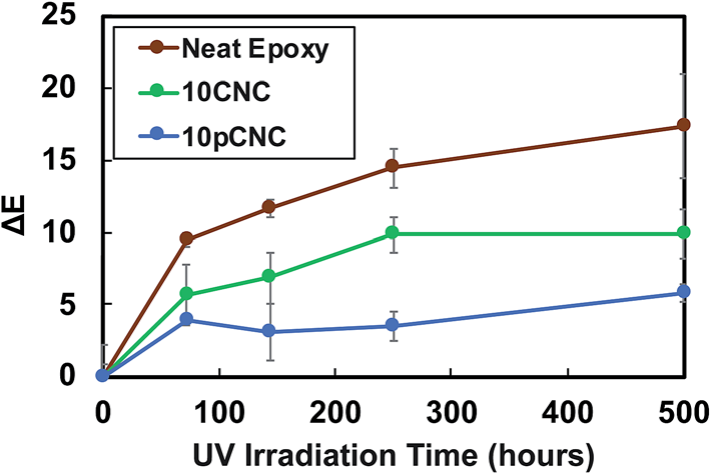
Discoloration of selected samples after exposure to UV radiation for a prolonged period of time.

To further investigate the impact of long term UV exposure on epoxy materials and the UV absorbance of pCNC, selected samples were exposed to UV radiation for extended periods of time (250 and 500 hours), and the discoloration was calculated and is presented in Fig. 9. The unfilled epoxy continued to discolor further with an increase in the UV irradiation time. In contrast, epoxies filled with 10CNC showed a lower rate of discoloration than the unfilled baseline epoxy. It was interesting to note that the irradiation of the 10CNC based epoxy nanocomposites for 250 and 500 hours did not present a statistically different color change, as shown in Fig. 9. The suspected reason for the UV absorption of CNC based nanocomposites was residual lignins left attached to CNCs during their manufacture, as lignin is a known UV blocker due to its phenolic, ketonic and other chromophore functionalities.^14,50^ The incorporation of pCNC at 10 wt% (10pCNC) substantially diminished the impact of UV-irradiation as observed from the lessened discoloration of the nanocomposites compared to both the unfilled and the 10CNC based nanocomposites. Moreover, the 10pCNC based nanocomposites displayed no color change beyond 144 hours of UV exposure. Hence, the grafting of PABA onto CNCs and their use as a UV protective molecule provided a very promising approach to mitigate discoloration and other property deterioration of epoxy as a result of UV degradation.

### 3.5. Thermal degradation of modified CNCs and the nanocomposites

**Fig. 10:**
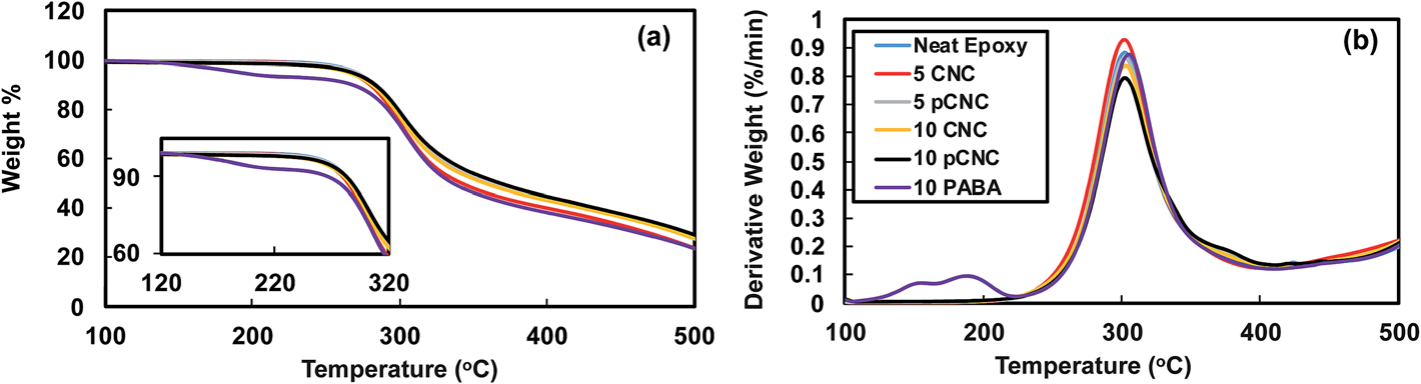
TGA analysis of epoxy nanocomposites with different fillers. (a) Weight loss curve and (b) weight loss derivative curve.

The thermal degradation behaviour of epoxy–CNCs was analyzed to investigate two main effects. First, to analyze the impact of incorporating CNCs, pCNC and PABA on the thermal stability of epoxy matrices. Second, to inspect the change in the thermal decomposition behaviour of the epoxy nanocomposites subsequent to subjecting them to UV-irradiation. The TGA thermograms of unfilled epoxy, and epoxy nanocomposites with CNCs and pCNC at 5 and 10 wt% loadings are presented in Fig. 10. For comparison purpose, the thermal degradation behavior of epoxy filled with PABA *via* direct addition was included in this study. The unfilled epoxy displayed a peak degradation at 302 °C. Such thermal decomposition of epoxy is mainly attributed to the dehydration caused by the elimination of water from the backbone structure of epoxy.^51^

The addition of CNCs and pCNC at both 5 and 10 wt% loading had a negligible impact on the thermal decomposition of the nanocomposites and the thermal stability was similar to that of the unfilled epoxy. Some literature reported that the incorporation of fillers such as clay at higher concentrations could result in deterioration of thermal stability of epoxy nanocomposites.^52,53^ The observation here with the addition of CNCs and pCNC at concentrations as high as 10 wt% did not cause a slump in the thermal stability of epoxy. This indicated that the CNCs (native and pCNC) did not impact the reactivity of epoxy with the amine curing agent and hence the crosslinking was maintained. Moreover, in all the CNC and pCNC nanocomposites a single degradation peak was observed suggesting that there was good interfacial interaction between the nanofillers and the epoxy. In contrast, the direct addition of PABA to epoxy resulted in a significantly reduced degradation onset temperature as shown in Fig. 10(a) and (b). As presented in Fig. 3, PABA itself has a low onset thermal degradation temperature. Thus, epoxy-nanocomposites with PABA also exhibited a lower onset degradation temperature as the PABA decomposes at lower temperature substantiated by the observation of a new derivative degradation peak at a lower temperature (180 °C) than for the rest of the epoxy nanocomposites. Thus, the covalent grafting of PABA onto CNCs was quite useful to overcome the thermal stability limitation of PABA for its use as a functional UV absorbing additive to epoxy and possibly other polymer systems.

### 3.6. Water absorption

**Fig. 11:**
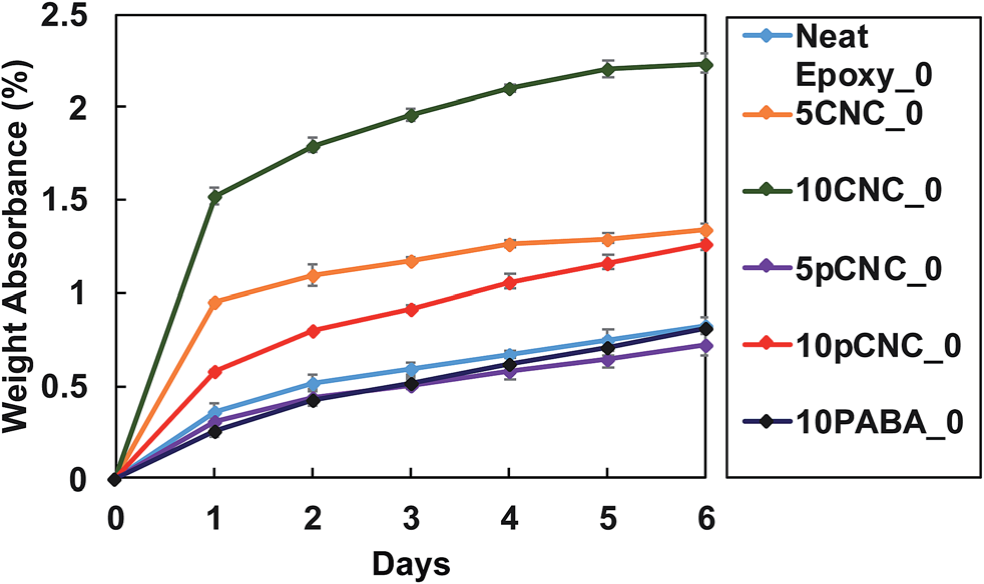
Water absorption of composite specimens prior to UV exposure.

Due to the inherent hydrophilicity of CNCs associated with their OH moieties, CNC–polymer nanocomposites typically display higher water absorption.^36^Fig. 11 presents the water absorption of the unfilled epoxy and its CNC and pCNC nanocomposites at 5 and 10 wt% loading. It was observed that as the immersion time increased the water uptake continued in all samples. The unfilled epoxy displayed a relatively low water uptake. However, the incorporation of CNCs led to a dramatic water absorption increase, and the increase was further exacerbated with the increase in the CNC loading from 5 wt% to 10 wt%. This was attributed to the hydrophilic nature of CNCs as shown in Fig. 4(a). The grafting of PABA onto CNCs (pCNC) substantially reduced the water absorption compared to the native CNC based nanocomposites. This was in line with the enhanced hydrophobicity of pCNC as observed from the WCA studies (Fig. 4). The increase in the loading of CNCs (native and pCNC) noticeably increased the water absorption. While a slowing trend in the water absorption kinetics was noticed with the increase in soaking time, equilibration was not achieved at the end of the seventh day.

## 4. Conclusions

In this study, tailored CNCs with excellent UV absorption properties were prepared by grafting a known UV filter molecule, *para*-aminobenzoic acid (PABA), onto CNCs in a two-step heterogeneous reaction by using a coupling agent that has differentiated reactivity (IPDI) among its reactive groups. The grafting of the UV filter molecule onto CNCs was confirmed by elemental analysis, IR and proton NMR spectroscopy analysis. The tailored CNCs exhibited enhanced thermal stability, hydrophobicity, and more importantly excellent UV absorption properties. Unfilled epoxies suffered from substantial structural change and severe discoloration under UV exposure due to mainly chain scission and chromophore formation, respectively. In contrast, epoxy polymers filled with the modified CNCs as a UV filter functional nanofiller displayed remarkable structural stability and less discoloration over a prolonged period of UV exposure. Additionally, the incorporation of pCNC maintained the thermal stability and reduced the hydrophilicity of epoxy nanocomposites which are beneficial for a range of material applications. In recent times a large amount of research has been conducted in using CNCs as a reinforcing filler to improve mechanical performance. However, CNCs have not been fully utilized to their full potential. This research focuses on one such functionalization of CNCs, modifying CNCs with PABA for UV-absorbing properties. The use of CNC–PABA as a general UV protective functional filler could be extended to other polymers that suffer from UV degradation. The potential application of modified CNCs is mainly in polymeric composites suffering from UV-degradation due to extensive exposure to UV light.

## Conflicts of interest

There are no conflicts to declare.

## Supplementary Material
